# Visualization of early influenza A virus trafficking in human dendritic cells using STED microscopy

**DOI:** 10.1371/journal.pone.0177920

**Published:** 2017-06-07

**Authors:** Faezzah Baharom, Oliver S. Thomas, Rico Lepzien, Ira Mellman, Cécile Chalouni, Anna Smed-Sörensen

**Affiliations:** 1 Immunology and Allergy Unit, Department of Medicine Solna, Karolinska Institutet, Stockholm, Sweden; 2 Genentech, Inc., 1 DNA Way, South San Francisco, CA, United States of America; Centre de Recherche en Cancerologie de Lyon, FRANCE

## Abstract

Influenza A viruses (IAV) primarily target respiratory epithelial cells, but can also replicate in immune cells, including human dendritic cells (DCs). Super-resolution microscopy provides a novel method of visualizing viral trafficking by overcoming the resolution limit imposed by conventional light microscopy, without the laborious sample preparation of electron microscopy. Using three-color Stimulated Emission Depletion (STED) microscopy, we visualized input IAV nucleoprotein (NP), early and late endosomal compartments (EEA1 and LAMP1 respectively), and HLA-DR (DC membrane/cytosol) by immunofluorescence in human DCs. Surface bound IAV were internalized within 5 min of infection. The association of virus particles with early endosomes peaked at 5 min when 50% of NP^+^ signals were also EEA1^+^. Peak association with late endosomes occurred at 15 min when 60% of NP^+^ signals were LAMP1^+^. At 30 min of infection, the majority of NP signals were in the nucleus. Our findings illustrate that early IAV trafficking in human DCs proceeds via the classical endocytic pathway.

## Introduction

Influenza A viruses (IAV) are responsible for causing a respiratory disease that is highly contagious and can escalate to life-threatening complications in vulnerable individuals [1]. The more pathogenic strains circulating during pandemics can also lead to death in healthy adults [2]. As obligate intracellular parasites, viruses are highly dependent on the host to replicate and infect new hosts [3]. Our immune system has evolved to protect us from invasion by pathogens such as IAV [4–6]. Among the first line of immune cells involved in surveillance, dendritic cells (DCs) have the superior ability to prime adaptive T cells with newly encountered antigens [7]. Concurrently, viruses have evolved immune evasion mechanisms to avoid recognition by immune cells [8, 9]. DCs arriving at the site of infection are susceptible to infection, and this may impair their ability to present antigens to CD8 T cells, important for viral clearance [5, 6, 10, 11].

The immune surveillance capacity of DCs is highly reliant on active endocytic processes [12]. Regulation of endocytic pH in DCs ensures that there is a balance between the destructive capacity of proteolytic enzymes and the ability to conserve antigenic peptides [13]. DCs control degradation of proteins by modulating the lysosomal pH to increase proteolysis upon maturation, induced after pathogen recognition [14]. This not only allows DCs to digest invading pathogens but also efficiently present them on their cell surfaces via major histocompatibility complex (MHC) molecules to activate T cells [15]. Similar to many other enveloped viruses, IAV are dependent on endocytic processes to penetrate the cellular membrane in a pH dependent manner [16]. Virions are sorted into vesicles and traffic through the endocytic machinery until arriving at the suitable endosomal compartment for fusion and release into the cytosol. Upon binding and internalization, IAV are sorted in endocytic vesicles and delivered to early endosomes that are characterized by proteins such as Early Endosome Antigen 1 (EEA1) and Rab5. The virus then progresses to late endosomes and lysosomes characterized by Lysosomal-associated Membrane Protein 1 (LAMP1) and Rab7 expression, where the pH is lower. IAV trafficking events have mostly been investigated in cell lines such as Madin-Darby Canine Kidney (MDCK) cells and Chinese Hamster Ovarian (CHO) cells, that are highly permissive to viral replication but may not be relevant in the context of an actual infection in humans [17, 18]. Nevertheless, these studies have elucidated the details of IAV entry pathway via a combination of biochemical techniques and electron microscopy. IAV require an acidic environment of pH ~5 (specifically depending on viral strain) in order to trigger conformational changes to the glycoprotein hemagglutinin (HA) that cause the viral and cellular membrane to fuse [19, 20]. Addition of a weak base such as NH_4_Cl is sufficient to inhibit viral replication due to a rise in pH to 6.7 [17]. More recently, there are studies involving human cell lines such as cervical epithelial HeLa cells and respiratory epithelial A549 cells also confirming the progress of IAV particles via early and late endosomal compartments prior to penetration into the cytosol and nuclear import, but at more delayed kinetics [21, 22]. Viral replication proceeds in the nucleus for IAV in a process known as “cap-snatching” where cellular RNA are used as primers to initiate its viral RNA synthesis [23]. Although DCs have been shown to be susceptible to IAV infection, it has yet to be investigated whether the virus traffics in a similar manner in these cells with a unique endocytic machinery [24].

Electron microscopy has been a long standing and valuable visualization technique by virologists to understand the morphology and infection pathways of viruses [18]. However, it is expensive, labor intensive and requires highly skilled personnel to ensure that there are no artifacts. In parallel, fluorescence microscopy has proved to be more accessible with the added benefit of being able to perform live cell imaging in order to reveal dynamic processes [25]. Furthermore, simultaneous assessment of multiple parameters is possible with fluorescence microscopy enabling colocalization studies. However, an obvious limitation in fluorescence microscopy is the resolution. There have been several efforts to overcome the diffraction limit, one of which is by Stimulated Emission Depletion (STED) microscopy [26–29]. This technique improves the point spread function by using a depletion laser, thus generating images with a resolution of approximately 40–50 nm in the X and Y axes, and 100 nm in the Z axis [30]. Super-resolution imaging provides new insight at the nanoscale level on the organization of cellular and viral proteins. In this study, we have capitalized on the improved resolution of STED imaging to visualize more accurately the early events of IAV trafficking in human DCs following internalization of virus particles.

## Methods

### Isolation and generation of dendritic cells

The use of blood from healthy blood donors was specifically approved for this study by the Regional Ethical Review Board in Stockholm, Sweden, and all experiments were performed according to the Declaration of Helsinki. Human MDDCs were differentiated as previously described [31]. In brief, buffy coats from healthy blood donors were obtained from the blood bank, Karolinska University Hospital, Stockholm, Sweden. Monocytes were obtained by negative selection using RosetteSep human monocyte enrichment cocktail (StemCell Technologies) followed by Ficoll-Paque Plus (GE Healthcare) gradient separation, and cultured with 40 ng/ml recombinant IL-4 and 40 ng/ml GM-CSF (both Peprotech) in complete medium referred to as R10 (RPMI 1640 (Sigma-Aldrich) supplemented with 10% fetal calf serum (FCS), 1% penicillin/streptomycin, 1% L-glutamine (all from Invitrogen) and 1% Hepes buffer (Sigma Aldrich). Medium was changed on day 3 and immature MDDCs were harvested on day 6. For incubations longer than 1 hour, MDDCs were cultured at 0.5x10^6^ cells/ml in R10 with IL-4 and GM-CSF.

### IAV infection of DCs

Influenza A/X31 (derived from Influenza A/aichi/2/68; H3N2) was propagated in chicken eggs, purified and concentrated on sucrose gradients (Virapur). Mock infected supernatants and allantoic fluid were processed in the same manner and used as controls to exclude any non-specific activation of DCs (data not shown). 50% tissue culture infective dose (TCID_50_) for IAV was determined by infecting a light monolayer of MDCKs in the presence of trypsin and monitoring the cytopathic effect. DCs were infected with 25 infectious particles (assessed in MDCK plaque assay) of IAV per DC (25 MOI). To synchronize entry of viral particles at early time points, DCs were adhered on Alcian-blue coated coverslips for 20 min and subsequently exposed to IAV at an MOI of 25 for 60 min at 4°C to allow virus binding to surface of DCs. After gently removing excess virus with PBS, DCs were transferred to 37°C and incubated for 0–30 min. To study later time points of viral infection, DCs were exposed to IAV for 4 hours before being adhered on coverslips. To prevent replication of IAV, 20 mM NH_4_Cl was added before addition of virus. All DCs on coverslips were fixed with pre-warmed 4% paraformaldehyde for 20 min at room temperature.

### Immunofluorescence

DCs adhered on coverslips were blocked with PBS containing 1% normal goat serum and permeabilized with 0.1% Triton-X 100 (Sigma) and stained with antibodies against IAV nucleoprotein conjugated to FITC (431/NP; Abnova), early endosome antigen 1 (14/EEA1; BD), lysosomal-associated membrane protein 1 (H4A3/LAMP1; BD) and HLA-DR conjugated to Biotin (L243; Biolegend). Secondary antibodies against mouse IgG conjugated to Alexa Fluor 555 (for confocal) and Alexa Fluor 532 (for STED), against FITC conjugated to Alexa Fluor 488, and Streptavidin conjugated to Alexa Fluor 647 (for confocal) or Alexa Fluor 568 (for STED) were used. Coverslips were mounted on glass slides with Prolong Diamond Antifade mountant (Molecular Probes) with DAPI (for confocal) or without DAPI (for STED). Confocal images were acquired on a Zeiss LSM700 using a 63x objective and STED images were acquired on a Leica SP8 STED 3X platform using a 100x white light, NA:1.4 oil immersion objective. To optimize resolution without bleaching, the STED lasers were applied at the lowest power that can provide sufficient improvement in resolution compared to confocal. Z-stack series were acquired in a specific order, beginning from the 568 nm laser line (STED laser: 660 nm), then 488 nm laser line (STED laser: 592 nm) and 532 nm laser line (STED laser: 592 nm) to ensure no bleaching of the other fluorochromes by the 592 nm STED laser. The quality of the Z-stack series was evaluated when optimizing the settings before acquiring all the images now included in the manuscript. STED images were deconvolved with Huygens Professional using the CMLE algorithm, with a signal to noise ratio (SNR) of 7 for HLA-DR and a SNR of 20 for the NP and EEA1/LAMP1 channels, at a maximum of 40 iterations. Full width at half maximum (FWHM) was determined by line profiles of fluorescence intensity across NP signals using ImageJ.

### Automated analysis of images using Python and scikit-image

To measure the degree of spatial coincidence between two or more staining signals, individual voxels were first classified as either positive or negative in each channel and connected positive voxels formed an object. Cell boundaries were defined based on the staining of HLA-DR. Images were first contrast enhanced and contours were detected with the marching squares algorithm. A whole cell was frequently not enclosed by a single contour, especially if cells displayed appendages. To consider cells as completely as possible, contours enclosing an area at least 20% as big as the most extensive contour were considered to be part of the most prominent cell in the image. This threshold was found by visual inspection to result in a good approximation of overall cell shape while being able to exclude cells which were only partially represented on an image. Based on the combined contours, a mask was generated which was used to determine whether identified features lay within the boundaries of a cell. NP or EEA1 positive pixels were identified by thresholding. A suitable threshold value was determined by careful visual inspection. To account for the diffuse character of stained LAMP1, a multi-step thresholding approach was chosen. Pixels were considered LAMP1 positive if their intensity was higher than the Otsu threshold multiplied by 0.8 and higher than the local mean intensity, or if their intensity was higher then the Otsu threshold multiplied by 1.5. Thresholding results were verified by visual inspection. For confocal image stacks, it was necessary to separate overlapping objects in the same channel using the watershed algorithm [32]. Colocalization was assessed by determining the proportion of shared voxels between objects. The overlap threshold for data acquired using a confocal microscope was set to 0.5, i.e. features were considered colocalized if 50% of all voxels for one object shared coordinates with another object. Given their higher resolution, colocalization analysis of STED data was performed using an overlap threshold of 0.1. The script was written in Python and utilized various libraries such as SciPy, NumPy and scikit-image.

### Statistical analysis

Data were analyzed using GraphPad Prism version 6.0 (GraphPad Software) and statistical significance was assessed using a paired t-test at 95% confidence interval. Data were considered significant at ** p<0.01.

## Results

### Improved resolution in visualization of viral trafficking in human DCs using STED microscopy combined with deconvolution

**Fig 1:**
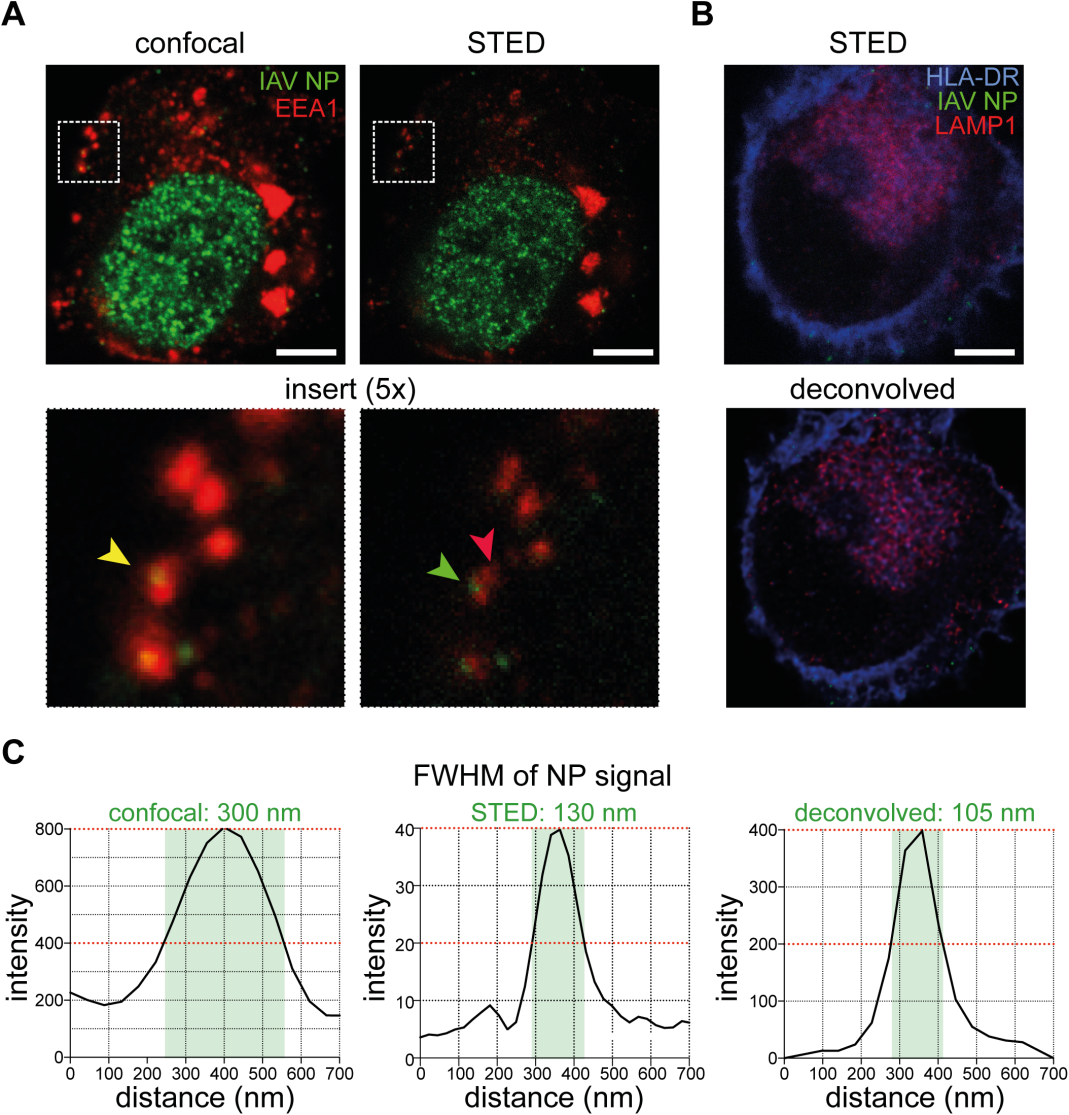
Improved resolution in visualization of viral trafficking in human DCs using STED microscopy with deconvolution. **(A)** Confocal (left panel) and STED (right panel) images of a DC 4 hours post infection with IAV, stained with antibodies against IAV NP (green) and EEA1 (red). Scale bar = 5 μm. **(B)** An image of a DC 0 min post infection with IAV, stained with antibodies against HLA-DR (blue), IAV NP (green) and LAMP1 (red) acquired by STED microscopy before (top panel) and after (bottom panel) deconvolution using Huygens Professional. Scale bar = 5 μm. **(C)** Full width at half maximum (FWHM) values of a representative NP signal from confocal, STED and deconvolved STED images was determined.

Influenza viruses rely on target cells to take up particles into their endocytic machinery for initiation of viral replication. This has been carefully characterized mostly in epithelial cell lines using a combination of biochemical methods and light or electron microscopy [18, 25, 33]. Although IAV infection typically affects the airways and the virus preferentially replicates in respiratory epithelial cells, human DCs are also susceptible to infection [11, 31]. As DCs specialize in preserving antigens for presentation to T cells and induction of pathogen-specific immunity, we hypothesized that they may handle viruses differently than other cells, as has been shown for HIV [34, 35]. Furthermore, membrane fusion of IAV is dependent on pH changes in the endosomal pathways to penetrate into the cytoplasm. The advent of super-resolution microscopy including STED has provided a novel method of performing multi-color 3D visualization of viral trafficking by overcoming the resolution limit imposed by conventional light microscopy without the laborious sample preparation of electron microscopy. To examine how the resolution is improved by STED, we exposed human *in vitro* monocyte-derived DCs (MDDCs) (S1 Fig) to IAV at a multiplicity of infection (MOI) of 25 for 4 h. The cells were then adhered to Alcian blue-coated coverslips, fixed and permeabilized before labeling with antibodies against the viral NP (green), EEA1 (red) and HLA-DR (blue). DCs were imaged in confocal mode, and then in STED mode by applying the depletion beams (Fig 1A). The representative image illustrates how distant signals of NP and EEA1 may appear to be overlapping when assessed by conventional confocal microscopy (Fig 1A, left panel) as the diffraction limit of about 200 nm is twice the size of a virus particle, whereas by STED imaging, the spatial overlap is now lost and visually, the NP and EEA1 signals are distinctly separated (Fig 1A, right panel). To better restore the resolution, 3D STED images were deconvolved using Huygens deconvolution software [36]. Indeed, with LAMP1 staining (red) that appears diffuse [37–39], the signal to noise ratio was improved after deconvolution to achieve better defined lysosomal structures (Fig 1B), allowing a more accurate assessment of whether the viral particles are spatially overlapping with different endosomal compartments. Quantification of the full width at half maximum (FWHM) values of a representative NP signal showed that the FWHM progressively decreased from confocal to STED and deconvolved STED (Fig 1C).

### 3D automated image processing and analysis of z stacks acquired by confocal or STED microscopy using scikit-image

**Fig 2:**
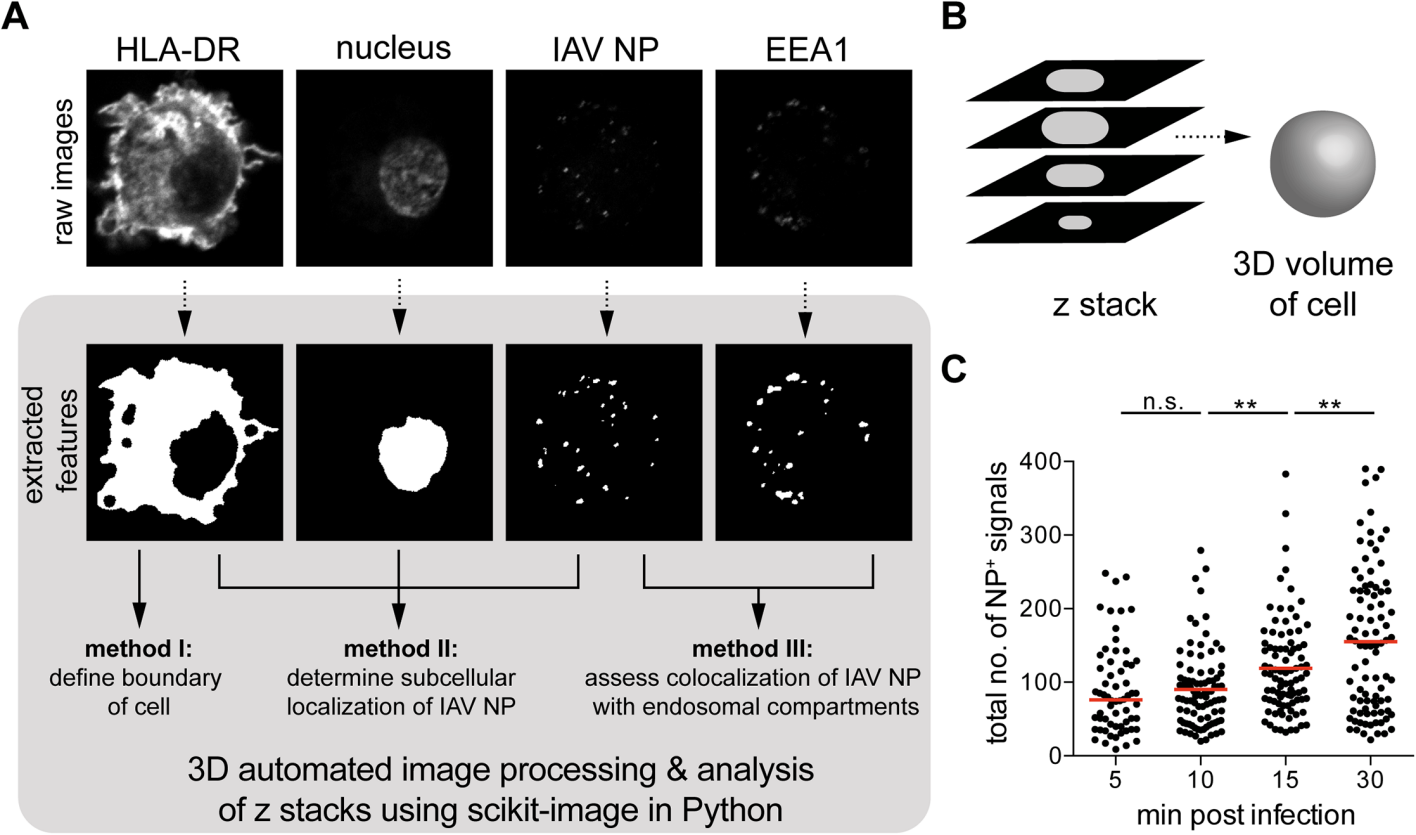
3D automated image processing and analysis of z stacks acquired by confocal or STED microscopy using scikit-image. **(A)** Raw microscope images of IAV-infected human DCs were processed to extract features such as the cell boundary, the nucleus, the viral particles and endosomal vesicles (method I). The extracted features were compared to each other using several methods to determine subcellular localization of IAV nucleoprotein (NP) (method II), or to assess colocalization of NP with endosomal compartments (method III). **(B)** Z stacks were analyzed as a whole to preserve the three-dimensional volume, taking into account overlapping features present in subsequent slices that may be counted repeatedly if slices were assessed individually. **(C)** The total number of NP^+^ signals in each volume of a cell was quantified (n = 60 cells per condition), with median values indicated by a red line. NP^+^ signals were not significantly different from 5 to 10 min, suggesting quantification of input virus, whereas increased significantly at 15 and 30 min, suggesting newly synthesized NP. Statistical differences were assessed using an unpaired *t* test: ** *p* < 0.01.

Here, we developed an automated system for high-throughput analysis using Python, a programming language, with image processing algorithms provided through the scikit-image package [32] that can provide unbiased analysis while still respecting the three dimensionality of acquired images. With this tool, we can isolate features from images (e.g. cell membrane or virus particles) and relate their spatial overlap with each other in a fully automated manner. Features from the raw channels of image files were extracted into a black-and-white format, thereby translating continuous intensity information into discrete binary information on the presence or absence of a particular structure at a voxel location; values in a three-dimensional (3D) space (Fig 2A). As z stacks were taken at a distance of 0.5 μm apart, we could maximize the spatial information on the localization of virus particles in the 3D volume of each cell (Fig 2B). Image data preprocessed in this manner was subsequently probed for spatial coincidence. The number of voxels shared by any two features was quantified and a threshold of 0.1 was set, specifying the minimum proportion of 10% shared voxels to consider them as colocalized. To assess the robustness of our analysis, we quantified the total number of NP^+^ signals (n = 60 cells per condition) and could report homogenous clusters of NP counts with few outliers (Fig 2C and Table A in S1 Table). Furthermore, the number of NP^+^ signals were not significantly different between 5 and 10 min, but began to increase significantly at 15 and 30 min, possibly due to increased separation of vRNP bundles allowing greater access for antibodies to bind to NP.

### Kinetics of IAV NP subcellular trafficking after being internalized by DCs

**Fig 3:**
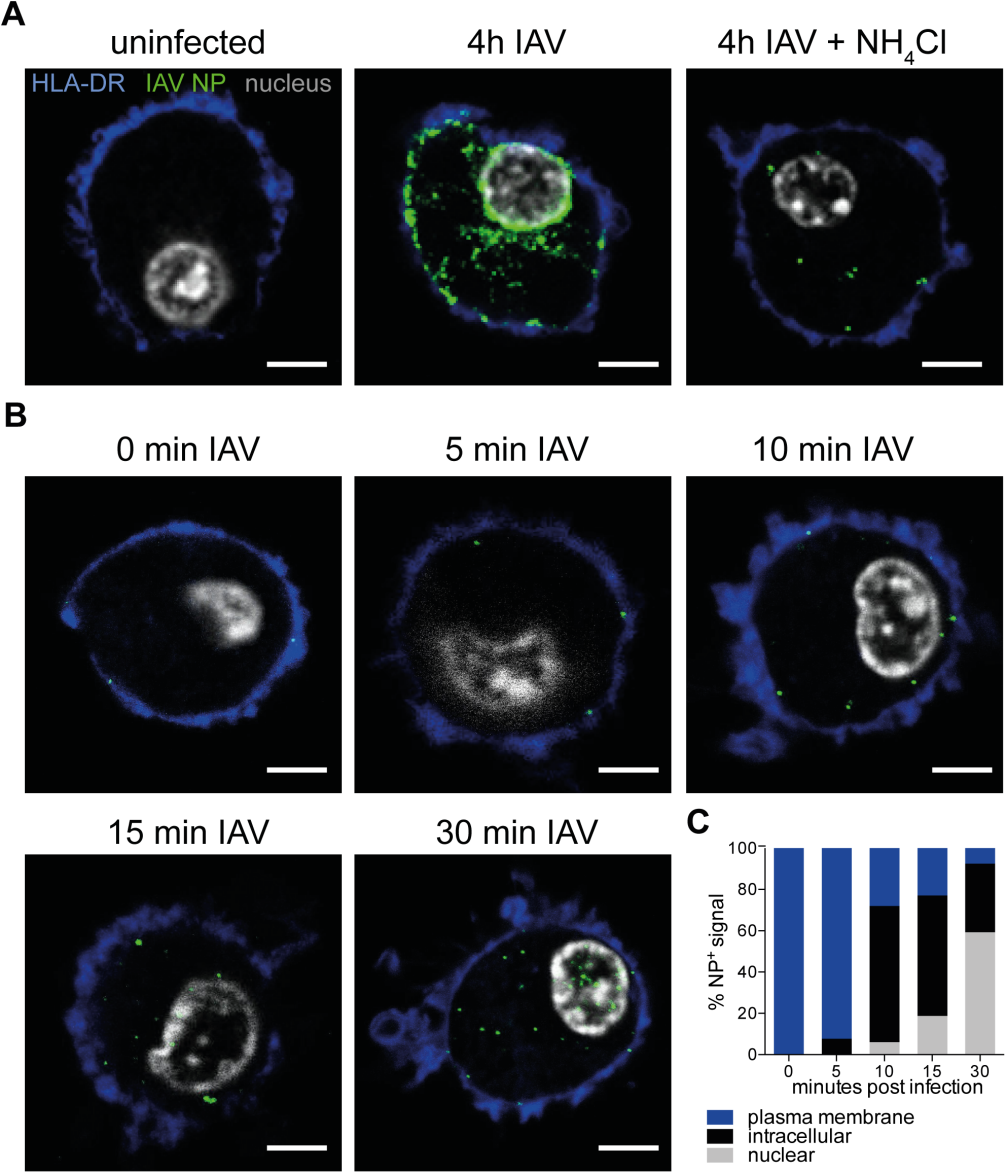
Kinetics of NP subcellular trafficking after entry in human DCs. **(A)** DCs were cultured with no virus, or infected with IAV for 4 hours in the absence or presence of NH_4_Cl. After 4 hours, cells were adhered on to Alcian blue-coated coverslips for 20 min and fixed with 4% PFA. DCs were blocked with 1% goat serum and permeabilized with 0.1% Triton X-100. DCs were labeled with primary antibodies against HLA-DR (blue), IAV nucleoprotein NP (green) and the nucleus was counterstained with DAPI (gray). All images were acquired by confocal microscopy on a Leica LSM700. Scale bar = 5 μm. **(B)** For earlier time points, DCs were first adhered to Alcian blue-coated coverslips for 20 min, exposed to IAV at an MOI of 25 for 60 min at 4°C to allow virus particles to attach to cell membrane, and incubated at 37°C for 0–30 min, allowing a more synchronized entry pattern. Scale bar = 5 μm. **(C)** The percentage of intracellular or nuclear NP^+^ signals relative to total NP^+^ signals in each volume of a cell was quantified using the Python script (n = 3 cells per condition). NP^+^ signals were in the nucleus as early as 10 min post exposure to IAV, with a majority of NP^+^ signals in the nucleus after 30 min.

To study the trafficking of IAV NP, we imaged the virus particles at different time points upon entering DCs in combination with markers for endocytic compartments. To serve as controls for identification of input and newly synthesized viral proteins, we cultured DCs for 4 h uninfected, or exposed to replicating virus in the absence or presence of NH_4_Cl, a weak base that prevents endosomal acidification hence limiting viral replication. DCs were stained with antibodies against HLA-DR (blue), IAV NP (green) while the nucleus was counterstained with DAPI (gray), and analyzed by confocal microscopy (Fig 3A). Viral NP was not detected in the uninfected controls, but several puncta could be observed in cells that were pre-treated with NH_4_Cl and exposed to IAV, suggesting that detection of input virus was possible. DCs exposed to IAV for 4 h showed large clusters of NP on the periphery of the nucleus, possibly in the Golgi/ER area, and also on the plasma membrane where assembly of virus particles for release could potentially be taking place. Next, we focused on earlier time points to understand the early events taking place after viral entry. To synchronize the entry of virus particles, we pulsed DCs with IAV at 4°C for an hour to allow viruses to bind to sialic acids on the cell surface, washed away excess virus and allowed bound virus particles to enter by incubating the cells at 37°C. Subsequently, cells were fixed at specific time points from 0 to 30 min (Fig 3B). Using confocal microscopy, viral NP was detected in intracellular compartments of human DCs already after 5 min with nuclear localization after 10 min. Finally, a majority of the NP signals was found in the nucleus after 30 min (Fig 3C). In summary, the data provided a time frame for us to assess the early trafficking events of IAV in human DCs between 0 and 30 min post infection.

### IAV particles traffic to EEA1^+^ early endosomes in DCs 5 min post exposure to IAV, and LAMP1^+^ late endosomes 15 min post exposure to IAV

**Fig 4:**
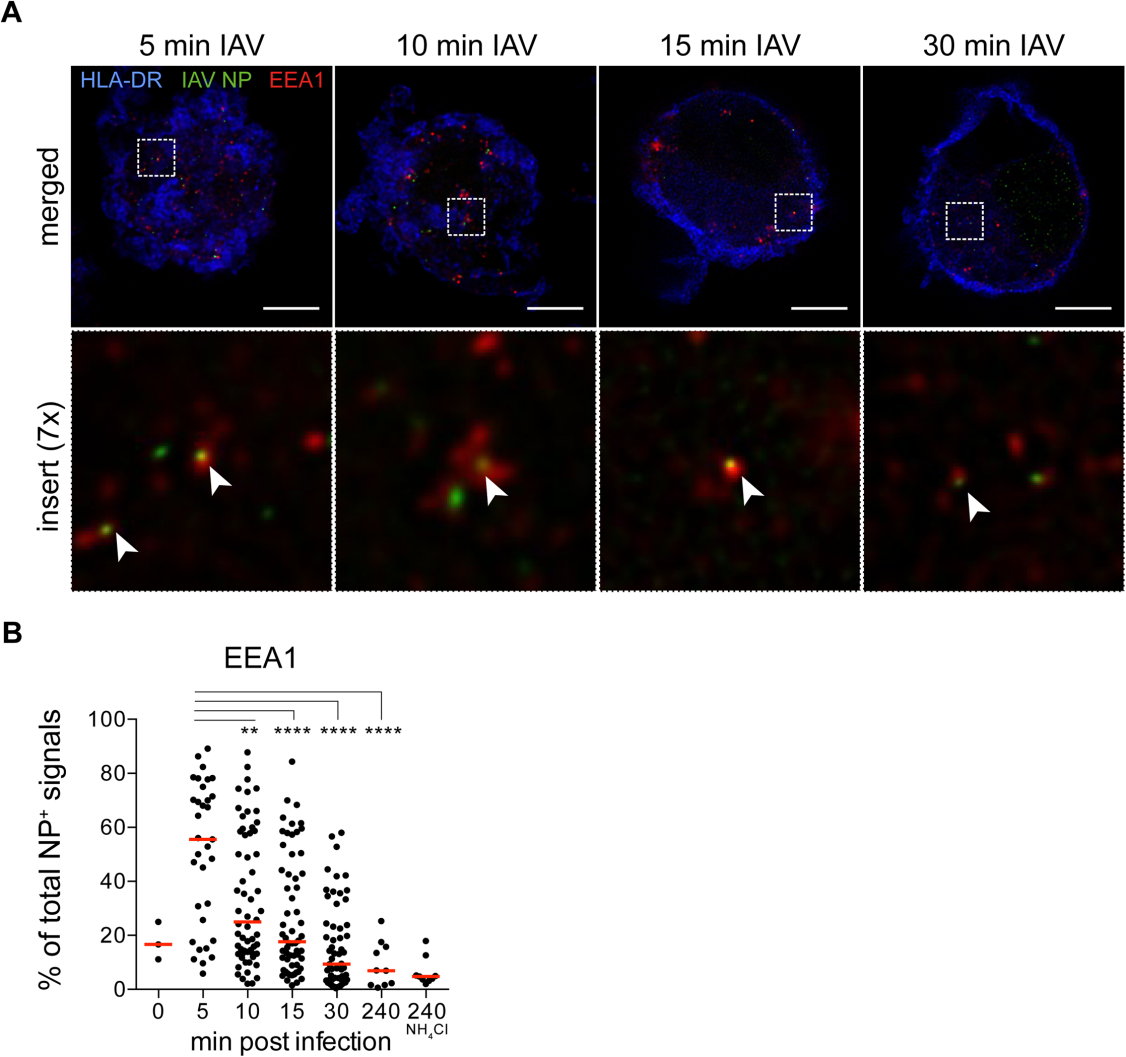
Trafficking of IAV particles to EEA1^+^ early endosomes in DCs occurred at 5 min post exposure to IAV. **(A)** DCs were labeled with antibodies against HLA-DR (blue), IAV nucleoprotein NP (green) and EEA1 (red). All images were acquired by STED microscopy on a Leica SP8. Images were deconvolved using Huygens Professional. Merged images of HLA-DR, IAV NP and EEA1 from one cell per condition and a insert at 7x magnification of NP and EEA1 are shown (n = 30 cells per condition). Arrow heads point to EEA1^+^ NP^+^ signals. Scale bar = 5 μm. **(B)** The percentage of NP^+^ signals in each volume of a cell also coinciding with EEA1^+^ signals out of total NP^+^ signals was quantified (n = 10–60 cells per condition) with median values indicated by a red line. EEA1^+^NP^+^ signals peaked at 5 min post infection. Statistical differences were assessed using an unpaired *t* test: ** p < 0.01, **** *p* < 0.0001.

**Fig 5:**
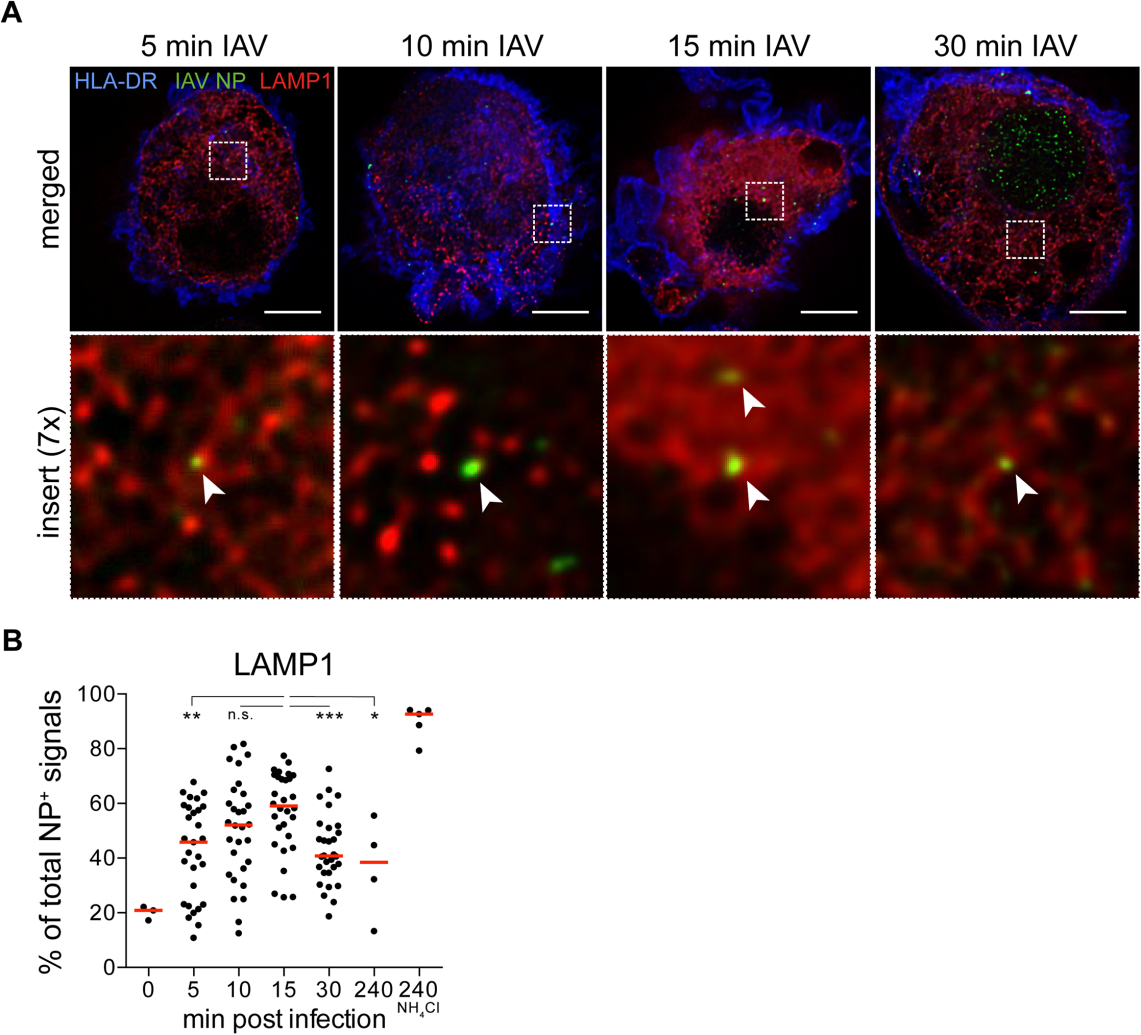
Trafficking of IAV particles to LAMP1^+^ late endosomes in DCs peaked at 15 min post exposure to IAV. **(A)** DCs were labeled with primary antibodies against HLA-DR (blue), IAV NP (green) and LAMP1 (red). All images were acquired by STED microscopy on a Leica SP8. Images were deconvolved using Huygens Professional. Merged images of HLA-DR, IAV NP and LAMP1 from one cell per condition and a insert at 7x magnification of NP and LAMP1 are shown (n = 30 cells per condition). Arrow heads point to LAMP1^+^ NP^+^ signals. Scale bar = 5 μm. **(B)** The percentage of NP^+^ signals in each volume of a cell also coinciding with LAMP1^+^ signals out of total NP^+^ signals was quantified using the Python script (n = 3–30 cells per condition) with median values indicated by a red line. LAMP1^+^NP^+^ signals peaked at 15 min post infection. Statistical differences were assessed using an unpaired *t* test: ** p < 0.01, *** p < 0.001, n.s., not significant.

**Fig 6:**
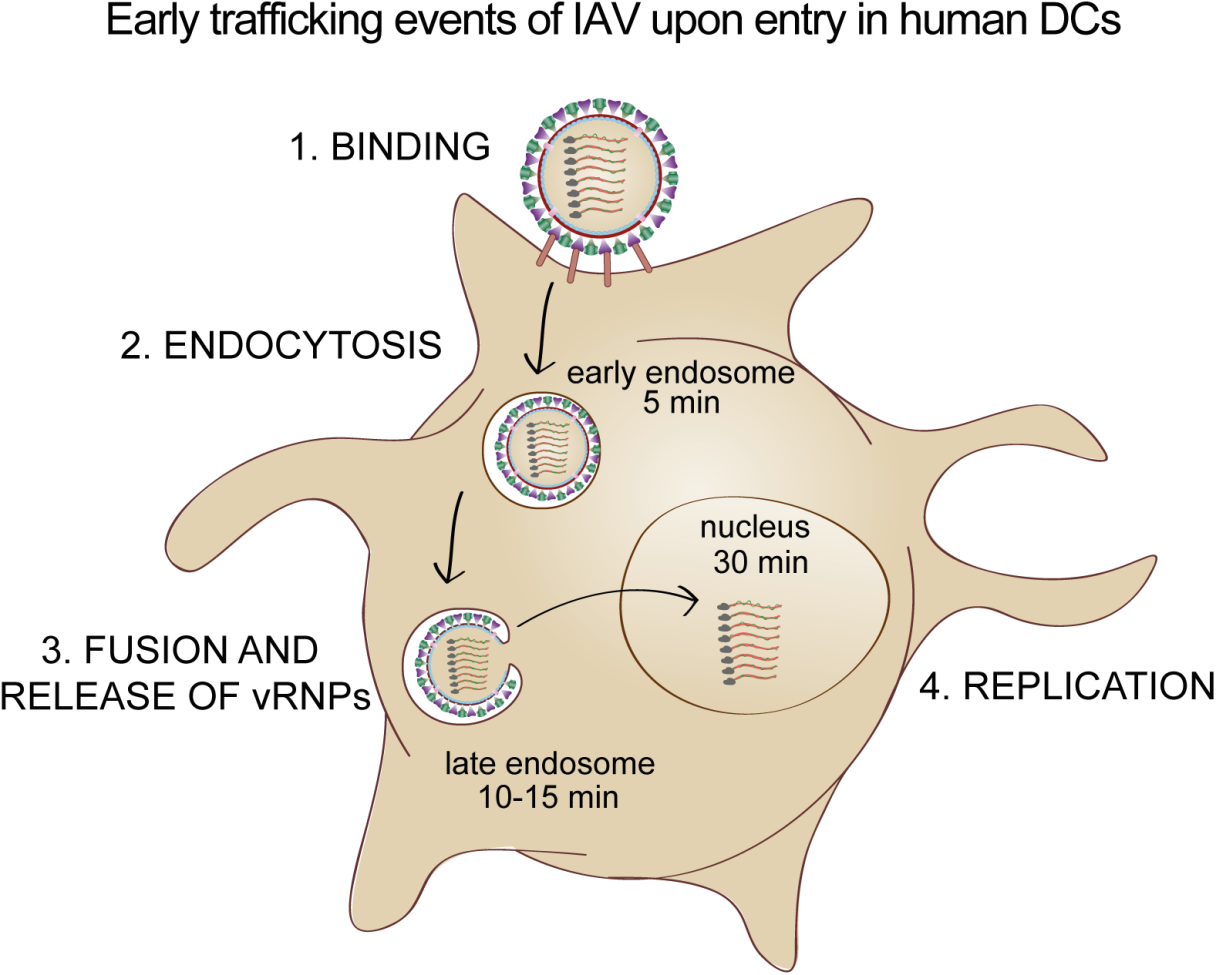
Early trafficking events of IAV upon entry in human DCs. The schematic summarizes the endosomal trafficking pathway of IAV upon entry in human DCs, beginning with binding of IAV to receptors on the cell surface. Endocytosed IAV were targeted to EEA1^+^ early endosomes within 5 min, followed by LAMP1^+^ late endosomes where membrane fusion could take place. Release of viral ribonucleoproteins (vRNPs) led to nuclear translocation where viral replication could proceed.

To expand our observations by confocal microscopy that input virus particles traffic within endosomal compartments in the first 30 min, we stained IAV-exposed DCs with an early endosomal marker EEA1 and acquired images by STED microscopy. Similar to previous reports, EEA1^+^ vesicles (red) appeared close to the plasma membrane, as defined by HLA-DR (blue) highly expressed on the surface of DCs (Fig 4A) [37–39]. NP (green) could be detected in EEA1^+^ compartments between 5 and 30 min after viral exposure. Our automated analysis showed that on average, more than 50% of NP^+^ signals were EEA1^+^ at 5 min, with the frequencies dropping to the lowest at 4 h post infection (Fig 4B and Table B in S1 Table). Next, we stained IAV-exposed DCs with late endosomal and lysosomal marker LAMP1 and acquired images by STED microscopy. LAMP1^+^ vesicles (red) appear throughout the cell except for the nucleus, and showed a vesicular pattern (Fig 5A). Our automated analysis indicated that on average, almost 60% of NP^+^ signals were LAMP1^+^ between 10–15 min, and the frequencies were reduced at 30 min (Fig 5B and Table C in S1 Table). In conclusion, by super-resolution STED microscopy, we could more accurately measure the spatial overlap between IAV NP and endosomal compartments of human DCs as detected by immunofluorescence than with conventional confocal microscopy, thus elucidating the early trafficking events of IAV in human DCs (Fig 6). The more delayed kinetics in human DCs compared to epithelial cells where IAV penetrate the cytosol at the perinuclear region after 8 min, is consistent with the intrinsic feature of DCs in preservation of antigens for presentation to T cells.

## Discussion

In this study, we demonstrated that IAV particles enter human DCs and traffic through early and late endosomes before gaining access to the cytoplasm. Although this mechanism has been previously investigated, most studies have utilized animal epithelial cell lines such as MDCK and CHO that are useful models as they are permissive to infection and easy to propagate and manipulate. Several studies have validated and built upon these observations in A549 human lung epithelial cell lines [40–42]. Following up on observations that human DCs reside along the respiratory tract and blood DCs may infiltrate the site during IAV infection, we hypothesized that IAV may traffic in DCs differently than in their target epithelial cells, as has been reported for other viruses such as HIV-1 [35]. HIV can subvert the intracellular trafficking machinery of DCs to facilitate its own dissemination to T cells [35]. Furthermore, infection of DCs with IAV has been reported to affect their ability to present antigens [11], hence a greater understanding of where and when the virus particles localize within the DC may reveal clues to how they impair antigen presentation. In DCs, endosomal compartments contain MHC molecules that can be loaded with viral antigens for presentation [12]. Additionally, Toll-like receptors that can recognize viral RNA such as TLRs 3, 7 and 8 also reside in endosomal compartments [43]. Recognition of viral RNA via TLRs triggers the maturation program of DCs enabling them to produce pro-inflammatory cytokines, upregulate co-stimulatory molecules, and migrate to draining lymph nodes, thus leading to the robust activation of T cells [44, 45]. As primary DCs especially from the respiratory tract are rare and inaccessible in humans, we have used *in vitro* MDDCs, resembling in vivo inflammatory DCs, as a model for investigating viral entry in human DCs [46].

An important and novel aspect of this study is the use of STED microscopy to improve the resolution of immunofluorescence signals of virus particles and endosomal compartments. The conclusions made regarding potential interactions of two molecules become increasingly accurate with improved resolution. As exemplified in Fig 1A, two separate proteins that are not interacting may appear to be spatially co-localized by conventional light microscopy due to its inability to discriminate between objects less than 200 nm apart. As with the more diffuse LAMP1 staining, applying a deconvolution algorithm to the STED images further refines specific endosomal structures. Imaging is a powerful qualitative tool to visualize subcellular events that would be completely overlooked by other methods such as flow cytometry. Additionally, imaging can also be a quantitative tool by characterizing the kinetics of events occurring within the cell. In our study, we captured intracellular viral trafficking events every 5 min in DCs exposed to IAV, and analyzed the cells in 3D by taking optical slices of each individual cell. As a consequence, a large data set of images were generated that had to be analyzed in an unbiased and efficient way. The availability of open source packages and modules provides a well-documented programming tool in Python that can be flexible to the specific needs of the user [32].

Our analyses demonstrate that when entry in human DCs is synchronized, IAV traffic to early endosomes at 5 min post entry, and to late endosomes at 15 min post entry, and is finally targeted to the nucleus at 30 min. Existing studies in MDCK and CHO cells described these events at a much faster rate, as viral membrane fusion with late endosomes occurred in the perinuclear region 8–10 min after binding, suggesting that nuclear import happens soon after [17, 25, 47]. In a more relevant model of human cells, HeLa cells were infected with IAV/WSN/33 at an MOI of 100–200, and IAV particles were observed to colocalize with early endosomes after 10 min and late endosomes after 40 min [21]. In a separate study using A549 cells infected with IAV/WSN/33, colocalization with early endosomes peaked at 45 min whereas colocalization with late endosomes peaked at 120 min [22]. The variability in kinetics of IAV entry between different cell lines suggests that there is host cell dependence in the general endocytic machinery that influences the rate of viral trafficking to the different endosomal compartments. Additionally, the use of different viral strains at different MOIs may also contribute to disparities in rate of viral trafficking. In HeLa cells, inefficiencies in the entry processes including rate of fusion and nuclear transport of viral ribonucleoprotein were suggested to contribute to the abortive nature of IAV infection in those cells [48, 49]. In future studies, the observations highlighted in our study could be further confirmed by staining for several other known markers of endosomal compartments such as the Rab proteins. Additionally, biochemical methods could be applied to assess protein-protein interactions between IAV NP and host endosomal proteins.

Continuous efforts are focused on dissecting the machinery of how IAV infection in DCs impairs antigen presentation on MHC I, whereas exposure to heat-inactivated virus permits efficient presentation of antigens to CD8 T cells [11]. The tools developed in this study offer a means of delving into the elaborate and highly coordinated processes taking place in a DC during an ongoing viral infection. By immunolabeling proteins involved in the MHC I processing machinery, differences between DCs exposed to replicating or heat-inactivated virus could be compared to uncover possible mechanisms of impairment [50]. Infecting DCs with genetically-engineered IAV lacking specific viral proteins is another strategy to identify which viral proteins are critical in the impairment of antigen presentation, similar to how NS1 has been identified to antagonize the interferon pathway in host cells [51]. Altogether, a more detailed knowledge of how IAV enters, infects and manipulates a human DC can aid to elucidate novel immune evasion strategies by IAV. In the future, this may allow us to design better therapeutic strategies against the virus.

## Supporting information

S1 Fig Phenotypic analysis of MDDCs by flow cytometry. **(A)** Dot plots show CD1a^+^ CD14^-^ MDDCs that also express CD11c and HLA-DR. One representative donor of 5 is shown. **(B)** MDDCs were left uninfected, exposed to replicating IAV in the absence or presence of NH_4_Cl, or exposed to heat-inactivated IAV for 24 h at an MOI of 0.6. Dot plots depict MDDCs stained for IAV NP and CD86. One representative donor of 5 is shown.
(TIF)

S1 Table Individual data points from quantitative analysis of STED images. **(A)** Total number of NP^+^ signals in MDDCs exposed to replicating IAV (corresponding to Fig 2C). **(B)** The percentage of NP^+^ signals in each volume of a cell also coinciding with EEA1^+^ signals out of total NP^+^ signals (corresponding to Fig 4B). **(C)** The percentage of NP^+^ signals in each volume of a cell also coinciding with LAMP1^+^ signals out of total NP^+^ signals (corresponding to Fig 5B).
(XLSX)
