# Supplementary figures and images for: Visualization of early influenza A virus trafficking in human dendritic cells using STED microscopy
Source: PLoS One. 2017 Jun 7;12(6):e0177920. doi: 10.1371/journal.pone.0177920 (PMC5462357; doi:10.1371/journal.pone.0177920)

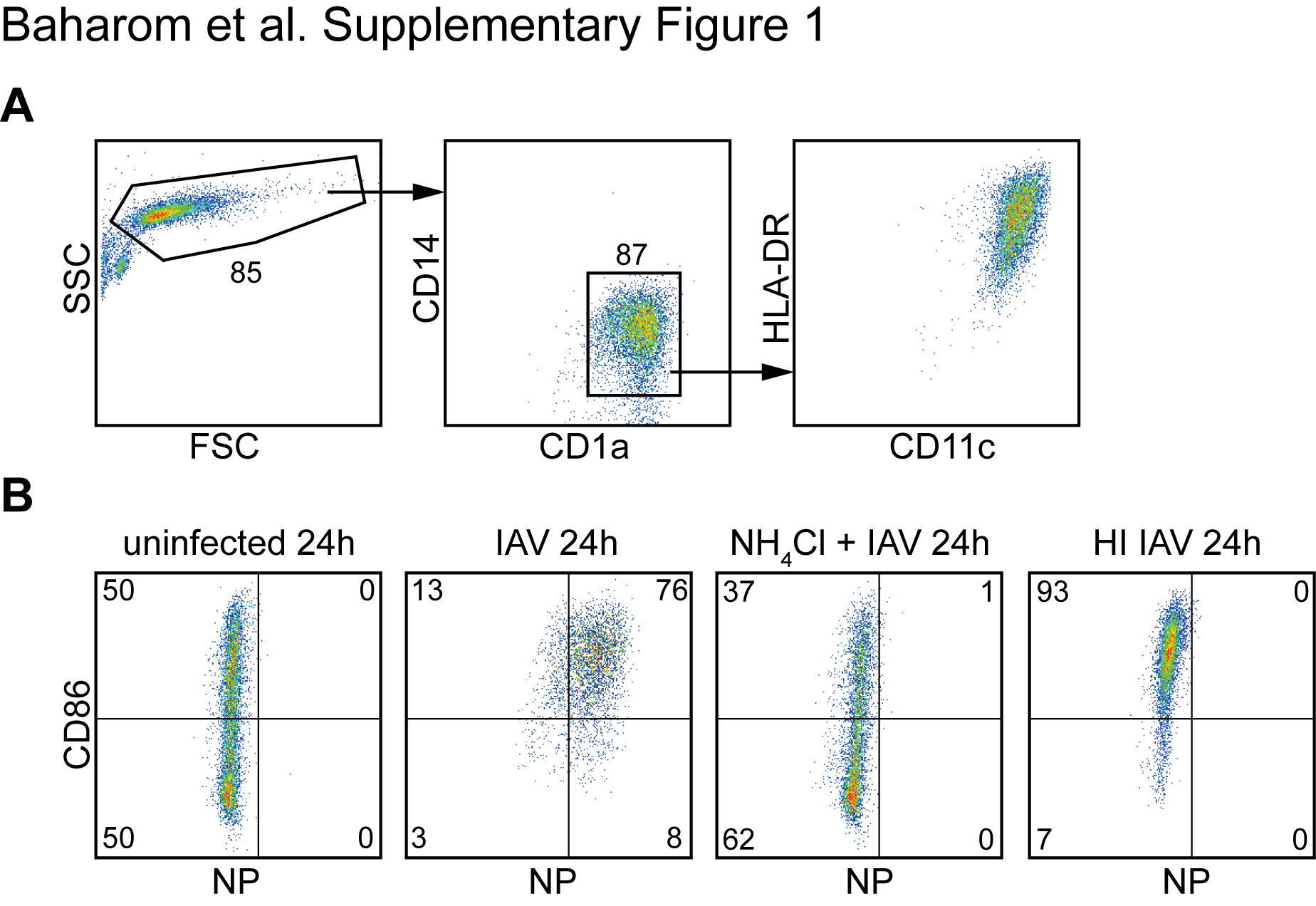

Supplement: S1 Fig — (A) Dot plots show CD1a+ CD14- MDDCs that also express CD11c and HLA-DR. One representative donor of 5 is shown. (B) MDDCs were left uninfected, exposed to replicating IAV in the absence or presence of NH4Cl, or exposed to heat-inactivated IAV for 24 h at an MOI of 0.6. Dot plots depict MDDCs stained for IAV NP and CD86. One representative donor of 5 is shown. (TIF) [file pone.0177920.s001.tif]
